# Unravelling the genetic architecture of cardiovascular disease through structural variant detection with whole-genome sequencing

**DOI:** 10.3389/fgene.2026.1747711

**Published:** 2026-02-25

**Authors:** Dona N. P. Colombage, Eric K. Moses, Phillip E. Melton

**Affiliations:** 1 Menzies Institute for Medical Research, University of Tasmania, Hobart, TAS, Australia; 2 School of Global and Population Health, The University of Western Australia, Perth, WA, Australia

**Keywords:** bioinformatics, cardiovascular disease, genome medicine, mobile elements, structural variants, whole genome sequencing

## Abstract

Cardiovascular disease (CVD) remains the leading cause of worldwide morbidity and mortality. Studies have found that there is a significant genetic component contributing to CVD development. Advances in genome sequencing technologies have revolutionized the identification of disease-causing variants in the human genome. With the development of whole genome sequencing (WGS), the understanding of these variants has been deepened as it enables comprehensive detection of many variants in the genome including structural variants (SVs). SVs are large genomic variants that are present in the genome of an organism and play a significant role in disease. Numerous techniques are being used to detect SVs with varying accuracy levels. Due to the limited number of focused research studies on SVs and CVD, there is a rich opportunity for further investigation with the aim of utilizing SV data in disease diagnosis and treatment plans. Emerging evidence highlights the role of SVs in CVD and the importance of adopting WGS approaches to unravel the genetic architecture of CVD. Moreover, integrating SV data with population scale epidemiology and advanced risk prediction models would enhance CVD prevention by enabling more personalized treatment strategies. This review aims to describe the different types of SVs and their involvement in CVD development and then to discuss WGS-based SV detection methods and future clinical implementations. We also report an overview of the SVs identified across various CVD types and different bioinformatics tools that can be used to detect SVs in WGS data.

## 1 Introduction

Cardiovascular disease (CVD) is an umbrella term that includes a range of conditions that impact the circulatory system. CVD is the leading cause of mortality worldwide, causing 32% of all global deaths (World Health Organization, 2021). CVD outcomes such as coronary artery disease, cardiomyopathy and heart failure have been found to have a 40%–60% significant genetic contribution to the disease risk (Drobni et al., 2022; Monte and Vondriska, 2014; Lindgren et al., 2018). While numerous genome-wide association studies (GWAS) have been carried out to identify and improve the understanding of underlying genetic variants to the risk of CVD, they have had various successes in pinpointing the specific genetic loci responsible (Monte and Vondriska, 2014).

GWAS have mainly focused on identifying common variants that show statistically significant association with a particular disease or a trait over a population (Golan et al., 2014). These common variants do not account for all the heritability due to factors including small effect size of individual common variants, exclusion of rare variants, gene-gene interaction (epistasis), epigenetics and the effect of environmental factors on genotype (Gibson, 2010). Furthermore, most of these studies have used single nucleotide polymorphisms (SNP) array data due to the logistics and cost of genotyping the large population sizes required for statistical power to detect disease associations, which leads to the under-examination of genetic regions which could significantly contribute to unexplained variance in disease risk (Tam et al., 2019; Chat et al., 2022). This problem was overcome by the introduction of Massive Parallel Sequencing (MPS). MPS technologies have revolutionized genomic research over the past 20 years by enabling the rapid, high throughput and cost-effective sequencing of DNA and RNA molecules (Satam et al., 2023). In particular, whole genome sequencing (WGS) has emerged as a promising method as it can detect nearly all the genetic variants in the genome in a comprehensive and unbiased manner (Chat et al., 2022).

WGS involves sequencing the entire genome of an individual to provide a thorough understanding of the genome. This technology allows the detection of genetic variants in non-coding, coding and regulatory regions of the genome, unlike targeted approaches like whole exome sequencing (WES) which primarily focus on the coding region. Advancements like improved resolution in WGS-based methods over WES and array-based methods have enabled researchers to detect structural variants (SVs) in the genome that may play a crucial role in the genetic contribution unexplained in the GWAS (Abel et al., 2020; Manolio et al., 2009).

SVs are large-scale genomic rearrangements that occur throughout the genome. These variants are more complex and diverse than SNPs and often span kilobase to megabase regions disrupting coding, non-coding and regulatory regions of the genome making them challenging to detect and interpret (Scott et al., 2021). Consequently, SVs have a higher impact on gene functions and regulations compared to SNPs (Scott et al., 2021; Wellenreuther et al., 2019). Traditional genomic research has prioritized SNPs due to their simplicity, abundance and ease of detection, which has diverted the attention and resources away from studying SVs. Therefore, SVs are relatively understudied even though they may have significant contributions to genomic variation which could contribute to many disease burdens including CVD (Wellenreuther et al., 2019; Feuk et al., 2006).

With the development of MPS technologies, WGS efforts in identifying SVs that are related to the pathogenesis of CVD have increased. WGS provides a powerful platform for identifying SVs due to its comprehensive coverage of the genome (Kosugi et al., 2019). This review will focus on SVs in the context of CVD, how the applications of short-read WGS-based strategies in CVD have revolutionized genomic medicine and the future clinical implications of SVs in CVD. Despite the advances enabled by WGS, much of our understanding of CVD genetics has historically come from GWAS. These studies provide essential context for why additional variant classes, particularly SVs, are needed to explain some of the remaining genetic variance that has not been captured by this approach.

### 1.1 Genome-wide association studies (GWAS) on cardiovascular diseases

These studies have provided new insights into the genetics that contribute to CVD development. GWAS aim to identify the genetic variants that are commonly associated with different diseases and traits by comparing variant frequencies between the cases and controls. More than a hundred hypothesis-free GWAS have been carried out over the past 15 years to identify the genetic loci associated with the CVD risk (Walsh et al., 2023). The finding of association between SNPs on 9p21 and coronary heart disease or myocardial infarction is the most strongly supported finding among different ethnic groups studied (Ndiaye et al., 2011). These studies were able to identify: a locus on chromosome 9p21 which was associated with coronary heart disease in Caucasian populations (McPherson et al., 2007), the SNPs in the genes *PHACTR1*, *SORT1*, *LDLR* and *PCSK9* are associated with coronary heart disease and myocardial infarction (Myocardial Infarction Genetics et al., 2009; Aragam et al., 2022), SNPs near lipid regulating genes such as *CYP7A1*, *NPC1L1*, *SCARB1, PPP1R3B and SLC39A8* lead to CVD risk factors such as higher total cholesterol levels in individuals with European ancestry (Teslovich et al., 2010; Waterworth et al., 2010), the mutations in *LDLR*, *PCSK9* and *APOB* genes cause familial hypercholesterolemia which is an inherited condition that result in higher cholesterol levels in the blood (Doi et al., 2021; Marmontel et al., 2023). Also, SNPs in genes *SH2B3*, *ATP2B1*, *CYP17A1*, *CYP1A2*, *MTHFR*, *ZNF652*, *PLCD3* and *FGF5* are associated with high blood pressure (Newton-Cheh et al., 2009; Levy et al., 2009). While GWAS have identified thousands of single nucleotide polymorphisms (SNPs) that are associated with CVD, most of these SNPs (more than 80%) reside in the noncoding region of the genome, therefore their functional significance remains unknown (Tam et al., 2019; Walsh et al., 2023; Ndiaye et al., 2011). While GWAS have been instrumental in identifying common variant associations, they capture only a fraction of the genetic architecture of CVD. This gap has motivated increasing interest in SVs, which can exert larger functional effects and may account for part of this unexplained variance.

## 2 Structural variations and cardiovascular diseases

**FIGURE 1:**
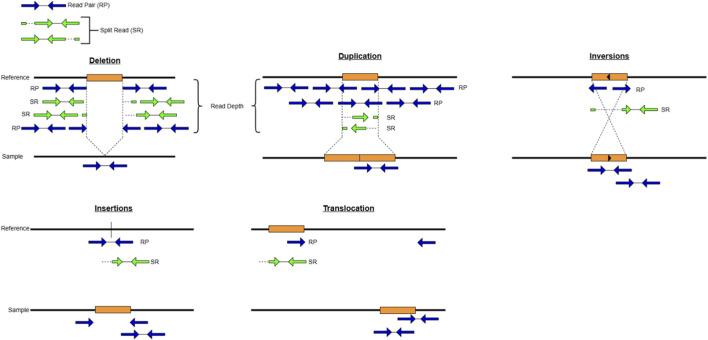
Types of SVS and approaches to detect SVS using paired-end sequencing data. Deletions can be detected using read depth (where deleted region has low coverage), read pair and split reads methods. Duplications can be detected using read depth (where the duplicated region has high coverage), read pair and split read methods. Inversions, insertions and translocations can be detected using read pair and split read methods where reads have unexpected orientations and insert sizes.

SVs are large genomic variants that exceed the length of 50bp (Ho et al., 2020; Collins et al., 2020). Different types of SVs include deletions, duplications, inversions, insertions and translocations (Figure 1; Table 1). These variants result in the genome due to different erroneous DNA replication, repair and recombination events (Escaramís et al., 2015). Activities of mobile elements (MEs) are also known to cause the formation of SVs in the genome (Steely et al., 2021). It has been found that SVs are responsible for genetic diversity in populations and due to their nature of affecting broader gene regions they contribute to numerous phenotypic variations and disease types (Abel et al., 2020; Ho et al., 2020; Sudmant et al., 2015). However, these variants are poorly studied and their involvement in CVD development is even under-explored and only very few studies are available (Table 2) (Chen et al., 2021).

**TABLE 1:** Summary major classes of SVs and commonly used detection tools.

SV type	Description	Commonly used short read SV detection tools	Notes
Deletion	Loss of genomic segment	DELLY, LUMPY, Manta, Breakdancer	Easy to detect using short reads
Duplication	Gain of genomic segment	DELLY, LUMPY, CNVnator	Easy to detect using short reads
Inversion	Genomic segment in reversed orientation	DELLY, LUMPY, Manta	Often copy neutral therefore no read depth signal. Short reads can detect inversions, but long reads increase breakpoint accuracy
Insertion	Insertion of novel genomic segment	Manta	Short reads have limited detection as large novel insertions and those occur in repetitive regions can be often missed but long reads can improve detection
Translocations	Rearrangement of genomic segments between nonadjacent regions or chromosomes	DELLY, LUMPY, Manta, Breakdancer	Short reads can detect with limited mapping clarity, but long reads provide clean breakpoint resolution as these frequently occur in repetitive or fragile genomic sites
Mobile elements	Genomic segments that can move	MELT, Mobster	Short reads can detect but long reads improve detection in repetitive regions

**TABLE 2:** SVs associated with different CVD outcomes.

SV type	Location/Gene	CVD type	Study type	Sample size	Validation method	Inheritance/Penetrance pattern	Functional studies	Other	References
Deletion	5q deletion (q35.1q35.3), *CSX*	Ventricular myocardial non compaction	Case study	One patient	FISH	NR	NR	Finding that requires further validation	Pauli et al. (1999)
Deletion	Del (chr 8) (p23.1), *GATA4*	Congenital heart disease	Case studies	Five (Pehlivan et al., 1999), three (Wu et al., 1996) and four (Wat et al., 2009) patients	FISH aCGH	NR	NR	Candidate variant supported by case studies	Pehlivan et al. (1999), Wu et al. (1996), Wat et al. (2009)
Deletion	chromosome 14, *MYH7* gene (exon 34–40) and the *MYH6* gene (exon 1–33)	Hypertrophic cardiomyopathy	Case study	One patient and family members	SNParray	Paternal inheritance	No	Finding that requires further validation	Mancuso et al. (2025)
Deletion	LMNA (exon 3–12)	Dilated cardiomyopathy	Case study	One patient	MLPA, qPCR and Immunostaining	NR	NR	Finding that requires further validation	Gupta et al. (2010)
Deletion	*MYBPC3* and *PLN*	Hypertrophic cardiomyopathy	Cohort study	387 unrelated HCM patients	MLPA, qPCR	NR	No	Candidate variant supported by cohort studies Pathogenic variant	Mademont-Soler et al. (2017)
Deletion	*BAG3* (exon 4)	Dilated cardiomyopathy	Family segregation	Three family members (proband, affected father and unaffected mother)	aCGH	Autosomal dominant	Yes	Finding that requires further validation	Norton et al. (2011)
Deletion	*TBX5* and *TBX3*	Holt–Oram syndrome	Case study	One patient	NR	NR	NR	Finding that requires further validation	Bogarapu et al. (2014)
Deletion	4q25 *PITX2*	Cardiac electrical and structural defects	Family segregation	7 families	PCR and Sanger sequencing	Autosomal dominant	Yes	Finding that requires further validation	Baudic et al. (2024)
Duplication	*TNNT2* and *LMNA*	Hypertrophic cardiomyopathy	Cohort study	505 unrelated HCM patients	aCGH	NR	NR	Finding that requires further validation	Lopes et al. (2015)
Duplication	chromosome 14q11.2, *MYH7* gene (exon 34–40) and the *MYH6* gene (exon 1–32)	Hypertrophic cardiomyopathy	Case study	One patient	qPCR	NR	NR	Finding that requires further validation VUS	Hamidi et al. (2025)
Deletion and Duplication	Chromosome 10, *CTNNA3* and chromosome 8 *GATA4*	Atrial fibrillation	Meta analysis	52,416 cases and 277,762 controls	RNA-seq, qPCR	10%–30% penetrance	Yes	Finding that requires further validation, Association signals	Choi et al. (2025)
Duplication, Inversion and SINE element	Chromosome 9p24 KANK1/DMRT1 loci	Congenital heart defect	Family segregation	Five generation family	Sanger, FISH and PCR	Autosomal dominant	NR	Finding that requires further validation	da Costa et al. (2024)
CNVs	*CDC73*, *DISC1*, *CDCP1*, *RET, PIK3C2G* and *CDH13*	Hyperlipidemia and myocardial infarction	Case-control study	31 cases and 9 controls	qPCR	NR	NR	Finding that requires further validation, Association signals	Shia et al. (2011)
CNVs (insertions-187, deletions-60)	​	Ischemic stroke	Genome wide analysis	263 cases and 275 controls	NR	NR	NR	Finding that requires further validation	Matarin et al. (2008)
CNVs	22q11.2, *GATA4*, *NKX2-5*, *TBX5*, *BMP*, and *CRELD1*	Congenital heart disease	Cohort study	167 patients	CNV-seq	NR	NR	Finding that requires further validation	Li et al. (2019)
CNVs	12p13.31, *SLC2A3*, *SLC2A14* and *NANOGP1*	Congenital heart defects	Cohort study	436 patients	qPCR	Autosomal and X-linked	No	Finding that requires further validation, Association signals	Prakash et al. (2016)
CNVs	*LPA*	Coronary artery disease	Case-control study	271 cases and 207 controls	NR	NR	NR	Finding that requires further validation, Association signals	Wu et al. (2014)
CNVs	*CYFIP1*, *NIPA1*, *NIPA2*, *DUSP1*, *JUN*, *JUP*, *MED15*, *MED9*, *PTPRE*, *SREBF1*, *TOP2A*, and *ZEB2*	Congenital heart disease	Case-control trio study	538 case trios and 1301 healthy control trios	ddPCR	*De novo*	NR	Finding that requires further validation	Glessner et al. (2014)
CNVs	22q11.2 region	Congenital heart disease	Cohort study	212 unrelated patients	FISH, CMA and ddPCR	Mostly *de novo*	NR	Finding that requires further validation Pathogenic and VUS variants	Zodanu et al. (2021)
CNVs	1q21.1, *GJA5*	Congenital heart disease	Case-control study	2436 cases and 6760 controls	MLPA	Variable penetrance	NR	Finding that requires further validation, Association signal	Soemedi et al. (2012)
Insertion	*BMPR2*	Heritable pulmonary arterial hypertension	Family segregation	152 patients	qPCR	NR	No	Finding that requires further validation	Kataoka et al. (2013)
Insertion	*ALMS1*	Alstrom syndrome	Case study	Two patients and their family members	NR	Autosomal recessive	NR	Finding that requires further validation	Taskesen et al. (2012)
Inversion	*CRFR1*	Heart failure	Case-control study	110 patients and 108 controls	qPCR	NR	Yes	Finding that requires further validation, Association signal	Pilbrow et al. (2016)
Translocation	Elastin locus	Supravalvular aortic stenosis	Family segregation	1 family	Restriction mapping and PCR	Autosomal dominant	NR	Finding that requires further validation	Curran et al. (1993)

The table lists the SV, types reported to be detected in relation to different CVD, types and their corresponding genes or genomic locations with the reference article. This table is a result of surveying the literature based on the SV, type detected and the CVD, type; FISH, fluorescent *in situ* hybridization; aCGH, array comparative genomic hybridization; MLPA, Multiplex ligation-dependent probe amplification; qPCR, quantitative polymerase chain reaction; CMA, chromosomal microarray analysis; ddPCR, digital droplet polymerase chain reaction; NR, not reported.

### 2.1 Copy number variations

Among the various classes of structural variation, copy number variants (CNVs) are the most extensively studied and represent a major contributor to dosage-related cardiac phenotypes. When the deletion, insertion and duplications cause variations in copy numbers compared to a reference genome these are called CNVs (Collins et al., 2020). Inversions and translocations do not cause CNVs in the genome (Collins et al., 2020). Deletions cause the loss of DNA segments from the genome; if they disrupt a coding region or regulatory region this can lead to a loss of function effect of a particular gene or several genes (Escaramís et al., 2015). Mademont-Soler et al. were able to identify and validate a *MYBPC3* deletion (entire exon 27 and a deletion spanning from exon 4 to exon 12) in 2 patients and a *PLN* gene coding region deletion in 2 patients out of 303 unrelated Spanish patients with hypertrophic cardiomyopathy (HCM) diagnosis (Mademont-Soler et al., 2017). Another genome-wide study was able to identify an 8733 bp deletion spanning the exon 4 of the *BAG3* gene in seven family members with dilated cardiomyopathy via comparative genomic hybridization (CGH) which was absent in 355 controls (Norton et al., 2011). Duplications result in the doubling of the genome regions, when the duplication occurs in the same region contiguously without any intervening sequence, it is called a tandem duplication (Van Dyke et al., 1983). A study by Lopes et al. was able to find a large deletion in the genes *MYBPC3* and *PDLIM3* and a large duplication in *TNNT2* and *LMNA* genes in 505 unrelated HCM patients using targeted sequencing methods focusing on the genes related to cardiomyopathy (Lopes et al., 2015). Recent studies have also shown the involvement of CNVs in CVD such as myocardial infarction (Shia et al., 2011), stroke (Matarin et al., 2008), coronary artery disease (Wu et al., 2014) and congenital heart diseases (Glessner et al., 2014). Different CNVs play a significant role in CVD in different populations and their frequency of occurrence may vary from one population to another (Pollex and Hegele, 2007). Overall, increasing evidence suggests that CNVs play an important role in CVD development, but larger population studies with WGS technologies will be required to better understand the clinical significance of these CNVs (Vijay et al., 2018). Beyond CNVs, structural variation also arises from mobile element activity, which generates insertions and complex rearrangements with important implications for CVD-related regulatory disruption.

### 2.2 Mobile elements (MEs)

**FIGURE 2:**
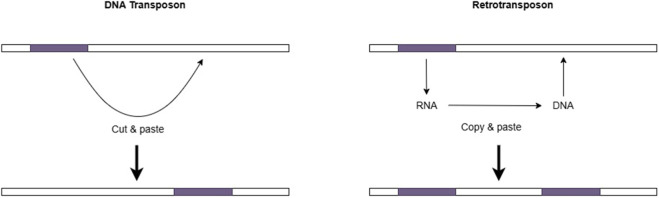
Two categories of MES: MEs can be separated into two main two categories based on their mobilization element. DNA transposons excised from one genomic location and mobilize using a cut and paste mechanism to integrate into another site. Retrotransposons mobilize using a copy and paste mechanism, where they transcribed into RNA and reverse transcribed into DNA before integrating into another site.

Insertion is an addition of a new DNA segment into the genome which may either disrupt genes or their regulatory regions, such as enhancers, silencers and topologically associated domains (TADs) (Escaramís et al., 2015). MEs are mostly responsible for inserting DNA fragments into the genome. These elements are repetitive DNA sequences capable of inserting themselves into different locations of the genome and contributing up to 42% of the human genome (Prak and Kazazian, 2001). MEs fall into two main categories (Figure 2): DNA transposons, which follow a ‘cut and paste’ mechanism to move and retrotransposons, which use an intermediate RNA molecule to move and will be then reverse transcribed to DNA before inserting into the genome. Retrotransposons can be further divided into long terminal repeats retrotransposons (LTR transposons) and non-LTR transposons which include long interspersed nuclear elements (LINES) (e.g., L1 element) and short interspersed nuclear elements (SINES) (e.g., Alu elements) (Prak and Kazazian, 2001). As these elements are heritable and dispersed throughout the genome, different loci could contain similar repetitive sequences susceptible to different mispairing and recombination events that lead to deletions or insertions in the genome (Kazazian, 1998; Xing et al., 2009; Kojima et al., 2023).

Studies have also shown the involvement of MEs in CVD development. Kataoka et al. found that exon 1 and 3 in the *BMPR2* gene have been deleted due to a recombination between two AluY elements in patients diagnosed with pulmonary arterial hypertension (Kataoka et al., 2013). Another study showed that the insertion of 333bp Alu Ya5 element in the *ALMS1* gene causes the Alstrom syndrome in a large Turkish family (Taskesen et al., 2012). Furthermore, the study of Holdt et al. was able to find that the Alu elements present in the *ANRIL* noncoding RNA, encoded by chromosome 9p21 which has been identified as the strongest genetic factor of atherosclerosis, are important in regulating the atherogenesis processes (Holdt et al., 2013). There are only very few studies that have been carried out to identify how MEs are involved in CVD development, therefore more investigations are needed to identify the effect of MEs on various CVD subtypes. In addition to ME-mediated events, several chromosome-scale rearrangements, including inversions and translocations, also contribute to structural complexity relevant to cardiovascular phenotypes.

### 2.3 Inversions and translocations

When a DNA segment breaks and is reattached to the same chromosome or different chromosome of the genome in an opposite direction it is called an inversion. This type of SV interferes with the chromosome pairing events during meiosis and potentially leads to recombination events (Kirkpatrick and Barton, 2006). With respect to CVD one study found that polymorphic inversion in chromosome 17q21.31 is involved in higher cardiac expression of *CRFR1* in heart failure patients (Pilbrow et al., 2016). Furthermore, few studies have also been carried out to identify the inversions that are related to CVD risk factors such as obesity (Gonzalez et al., 2020; Caceres and Gonzalez, 2015) and diabetes (Antonacci et al., 2009). When a DNA segment changes its location interchromosomally or intrachromosomally, it is called a translocation (Escaramís et al., 2015). The translocation can also cause disruption or dysregulation of genes depending on the chromosomal breakpoint. For example, Curran et al., identified a translocation that disrupts the elastin gene on chromosome 7 which is associated with the development of supravalvular aortic stenosis disease (Curran et al., 1993). They were also able to identify a 300bp Alu repeat within the intron 27 of the elastin gene when they were analyzing exon 28, which was the breakpoint of this translocation, suggesting that Alu elements may have been involved in the translocation mechanism (Curran et al., 1993). Generally, all the above variant types generate two breakpoints in the genome and, different methods are being used to identify these breakpoints and characterize the SV type.

## 3 Detection of structural variants

Detecting SVs presents several challenges due to their complexity and diversity. There are different types of SVs which vary in size, and they tend to occur in complex genome locations like centromeres and telomeres which have higher repetitive regions making their detection very difficult; therefore, many methods cannot cover all the types (Collins et al., 2020). Even though various techniques have been developed to detect SVs, each of the methods has its own strengths and limitations with no method on its own able to detect all SV types. In cytogenetics methods, SVs must be very large to be visible under the microscope and they cannot detect the breakpoints of SVs and complex SVs (Yang, 2020). The fluorescent *in situ* hybridization (FISH) and comparative genomic hybridization (CGH) methods can detect only the copy number changes even though there are many other types of SVs. The low sensitivity and the low precision of the above methods have been overcome with the development of MPS techniques (Yang, 2020). These methodological challenges highlight the need for sequencing technologies capable of resolving SVs with higher precision. WGS, especially emerging long-read platforms, directly addresses several of these limitations.

### 3.1 Structural variants and whole genome sequencing

Many SVs can exceed the length of the short reads generated by ‘short read’ WGS technologies, thereby increasing the difficulty of detecting SVs and SVs present in the repetitive regions (Escaramís et al., 2015; Mahmoud et al., 2019; Liu et al., 2024). The introduction of ‘long read’ WGS technologies has overcome this read length challenge and has the advantage of detecting complex SVs and SVs in repetitive regions over the other methods (Yang, 2020; Liu et al., 2022). Since short reads are preferred over long read WGS technologies for larger data sets and population data research due to their relatively low cost and high throughput, to facilitate the identification of SVs using short reads four main conceptual strategies have been developed.

These include Read Depth, Read Pair, Split Read and Assembly Methods. The Read Depth (RD) method identifies the unexpected changes in the read count (depth of coverage) in the genomic regions and is able to detect copy number variants but is limited in breakpoint detection (Yang, 2020; Zhao et al., 2013). The read pair (RP) method utilizes the orientation, and the span of paired-end reads to identify the inconsistencies between the reference genome and the read pairs, but it is known to be less effective in repetitive regions (Alkan et al., 2011). The split read (SR) method detects the paired reads where one read mapped completely to the reference genome while the other read fails or is only partially mapped to the reference; the reads that fail to map are used to identify the breakpoint of SVs (Jakubosky et al., 2020). The assembly method (AS) generates contigs by *de novo* assembling the reads and the contigs are then mapped to the reference genome to identify the SVs (Guan et al., 2016). The assembly method can detect more SVs compared to other methods, but it is limited in use due to its high demand for computational resources (Alkan et al., 2011). Taken together, these strategy-specific strengths and limitations underscore the importance of evaluating SV discovery holistically rather than tool-by-tool.

Across SV discovery workflows, three recurring constraints determine downstream interpretability: (i) repeat content and reference bias, (ii) breakpoint precision, and (iii) cohort scalability. Short-read pipelines (RD/RP/SR/AS) remain cost-efficient and scalable for population studies yet systematically underperform in repeat loci and in detecting novel insertions, leading to ambiguous breakpoints that weaken mechanistic inference and reduce association power across cohorts (Ho et al., 2020; Mahmoud et al., 2019). Long-read and HiFi approaches mitigate these issues with improved breakpoint resolution and enhanced detection in complex or repetitive regions but are still limited in large biobank-scale studies due to cost and throughput (Yang, 2020; Liu et al., 2022). These trade-offs motivate hybrid strategies, such as discovery using short reads followed by targeted long-read or optical validation (Escaramís et al., 2015; Liu et al., 2024). At the caller level, combining multiple evidence types, RD for CNVs, RP for insertions/inversions, SR for precise breakpoints, and AS for complex alleles, consistently outperforms single tools, provided merging uses calibrated confidence thresholds and orthogonal validation matched to SV class/size (Kosugi et al., 2019; Alkan et al., 2011). These methodological choices directly shape clinical translation, influencing gene-level attribution, pathogenicity classification, and diagnostic yield (Pagnamenta et al., 2023; Stranneheim et al., 2021) as well as epidemiological analyses, where breakpoint uncertainty and reference bias affect genotype concordance, and allele frequency estimation (Collins et al., 2020; Sudmant et al., 2015). Building on these conceptual approaches, a diverse ecosystem of SV callers has been developed, each leveraging different combinations of RD, RP, SR, or assembly signals to optimize detection across SV types.

### 3.2 Bioinformatic tools

Numerous bioinformatics tools have been developed to detect SVs in the sequencing output data (Table 3). Some tools are specialized in detecting SVs in tumor samples and some are specialized in the input data types such as ‘short read’ and ‘long read’ sequences. Every tool shows different levels of precision and recall rates in calling SVs of different types, different sequencing depths and different SV size ranges (Kosugi et al., 2019; Mahmoud et al., 2019).

**TABLE 3:** SV callers.

Tool	SV type	Method	Sequencing type	Use case	Input/Output	Strengths and limitations	Computational footprint	URL
BIC -seq	CNV	RD	Short reads	Somatic and germline CNV	BAM to CNV segments (.seq)	high sensitivity of detection for small CNVs, limited in breakpoint detection	Low-moderate	https://github.com/ding-lab/BICSEQ2
Canvas	CNV	RD	Short reads	Germline and paired tumor/normal	BAM to VCF	limited in breakpoint detection	Moderate	https://github.com/Illumina/canvas
Control-FREEC	CNVs and allelic imbalances	RD	Short reads (WGS/WES)	Somatic, germline, overdiploid tumor samples and samples contaminated by normal cells	SAM/BAM	corrects for GC-content and mappability biases, define the program’s behavior in low mappability regions	Low-moderate	https://github.com/BoevaLab/FREEC
CNVnator	CNV	RD	Short reads	Somatic, germline, family and population	SAM/BAM to VCF	high genotyping accuracy, misses CNVs created by retrotransposable elements	Low	https://github.com/abyzovlab/CNVnator
CNVseq	CNV	RD	Short reads	Germline	FASTQ,BAM to CNV segments	Can be applied to samples with low sequencing coverage	Low	https://github.com/sanadamakomi/CNVseq
MrCaNaVaR	CNV	RD	Short reads	Germline	SAM/BAM	Can detect large segmental duplications, works only with mrFAST or mrsFAST mappers	Low-moderate	https://github.com/BilkentCompGen/mrcanavar
cn.MOPS	CNV	RD	Short reads	Germline population	FASTQ to CNVs	Able to control the false discovery rate	Low-moderate	http://www.bioinf.jku.at/software/cnmops/
ReadDepth	CNV	RD	Short reads	Germline	BAM	Enable parallel processing	Low	https://github.com/chrisamiller/readDepth
CNAnorm	CNV	RD	Short reads	Tumor samples	SAM/BAM	Better normalization	Low	https://github.com/bioc/CNAnorm
SegSeq	CNV	RD	Short reads	Tumor samples	BAM	Precise breakpoint detection	Low	https://mybiosoftware.com/tag/segseq
CNVer	CNV	RD,RP	Short reads	Germline population	FASTQ	limited in breakpoint detection, cannot detect novel insertions	Low-moderate	https://github.com/pashadag/Cnver
CNVeM	CNV	RD, RP	Short reads	Germline population	BAM	Can detect CNVs at nucleotide resolution	Low-moderate	https://github.com/riyasingh07/CNVeM
BreakDancer	Del, Ins, Inv, Tra	RP	Short reads	Family/population germline, somatic	BAM to VCF	Fast, sensitive and accurate indel detection	Low	https://github.com/genome/breakdancer
Cloudbreak	Del, Ins	RP, Hadoop	Short reads	Germline population	FASTQ/BAM to	Big-data analysis	Moderate-high	https://github.com/cwhelan/cloudbreak
SVMiner	Del, Inv	RP, clustering	Short and long reads	Germline	BAM to VCF	Higher accuracy, not suited to detect larger deletions	Moderate	http://cbc.case.edu/svminer/
PEMer	Del, Ins, Inv	RP	Short reads	Germline	BAM	Limited in breakpoint detection	Low	http://sv.gersteinlab.org/pemer
RetroSeq	ME	RP	Short reads	GermlineME focused	BAM to VCF	Accurate ME calls	Low	https://github.com/tk2/RetroSeq
splazerS	Del, Ins	SR	Short reads	Germline	BAM	High precision and high sensitivity	Moderate	https://www.seqan.de/apps/
Pindel	CNV, Inv	SR	Short reads	Germline	BAM to VCF	Precise breakpoint detection	Moderate	https://github.com/genome/pindel
Gustaf	Del, Inv, Dup, Tra	SR	Short reads	Germline	BAM to VCF	Correctly classify SVs	Moderate	http://www.seqan.de/projects/gustaf/
SOAPSV	Del, Ins, Inv	AS	Short reads	Germline	FASTQ/BAM	Low false positive and false negative rates	Moderate	https://github.com/chienql/soap
Delly	CNV, Tra	RP, SR, RD	Short and long reads	Germline and somatic	BAM to VCF	Widely used, sensitive and accurate	Moderate	https://github.com/dellytools/delly
PRISM	Del, Ins, Inv, Dup	RP, SR	Short reads	Germline	BAM to VCF	Faster run time	Moderate	http://compbio.cs.toronto.edu/prism
GRIDSS	Del, Dup	RP, SR, AS	Short reads	Germline and somatic	BAM to VCF	Better breakpoint resolution	Moderate-high	https://github.com/PapenfussLab/GRIDSS
tangram	ME	RP, SR	Short reads	Germline population ME focused	BAM to VCF	Fast and memory efficient	Low-moderate	https://github.com/jiantao/Tangram
MELT	ME	RP, SR	Short reads	Germline population ME focused	BAM to VCF	Commonly used	Moderate	https://melt.igs.umaryland.edu/
inGAP-sv	Del, Ins, Inv, Tra	RP, RD	Short reads	Germline population	SAM to VCF	Significantly reduce false discovery rate, supports parallel computing	Moderate	http://ingap.sourceforge.net/
GASVPro	Del, Inv	RP, RD	Short reads	Germline	BAM to VCF	Slow	Low-moderate	https://docs.rc.ufl.edu/software/apps/gasvpro/
Genome STRiP	Del	RP, RD, AS	Short reads	Germline population and tumor	BAM to VCF	Best for cohort CNV analysis	Moderate-high	https://github.com/yigewu/genomestrip
Manta	Del, Ins, Inv, Dup, Tra	RP, SR, AS	Short reads	Germline and somatic	BAM to VCF	Commonly used, fast and sensitive	Moderate	https://github.com/Illumina/manta
Meerkat	Del, Ins, Inv, Dup, Tra	RP, SR, RD	Short reads	Somatic	BAM to VCF	Slow	Moderate	http://compbio.med.harvard.edu/Meerkat/
Lumpy	Del, Dup, Inv	RP, SR, RD	Short reads	Germline and somatic	BAM to VCF	Faster and low false positive rate, commonly used	Moderate	https://github.com/arq5x/lumpy-sv
TIDDIT	Del, Dup, Inv, Tra	RP, SR, RD, AS	Short reads	Germline, somatic	BAM to VCF	Fast	Moderate	https://github.com/SciLifeLab/TIDDIT
cnvHitSeq	CNV	RP, SR, RD	Short reads	Germline	BAM to VCF	Limited in breakpoint detection	Low	https://github.com/CoinLaboratory/cnvHitSeq
ERDS	CNV	RD	Short reads	Germline	BAM to VCF	Integrate SNP info	Low -moderate	https://github.com/igm-team/ERDS
Cue	Del, Inv, Dup	Deep learning	Short and long reads	Germline and somatic	BAM/CRAM to VCF	Accuracy depends on the training dataset	Moderate	https://github.com/PopicLab/cue
xTea	ME	SR and Supervised Machine learning approach	Short and long reads, barcode linked-reads and hybrid data from different sequencing platforms	Germline and somatic ME focused	BAM to gVCF	Faster analysis, Higher ME accuracy	Moderate-high	https://github.com/parklab/xTea
DeepMei	ME	Deep learning	Short reads	Germline population ME-focused	BAM to VCF	Faster analysis	Moderate-high	https://github.com/xuxif/DeepMEI

SV, type; CNV, copy number variation; Del–deletion; Ins–insertion; Inv -inversions; Tra–translocation; Dup–duplications; ME, mobile elements.

Method: RD, read depth; RP, read pair; SR, split read; AS, assembly method.

RD based tools such as CNVnator (Abyzov et al., 2011) and Canvas (Roller et al., 2016) and RP and SR based methods like Delly (RP, SR) (Rausch et al., 2012), LUMPY (RD, RP, SR) (Layer et al., 2014), Manta (RP, SR) (Chen et al., 2016) are widely being used for identifying SVs in WGS data. In a study to identify *de novo* variants and novel candidate genes related to congenital heart disease, Manta and Canvas tools were used to call CNVs (Hartill et al., 2024). In addition to the above approach-based tools, tools based on machine learning are also being developed to overcome the problems associated with approach-based methods such as caller-specific tools, problems associated with the sequencing properties and alignment algorithms. Cue (Popic et al., 2023) DeepSV (Cai et al., 2019) and SVision (Lin et al., 2022) are a few examples of SV calling tools, DeepSVFilter (Liu et al., 2021) is a false positive SV filtering tool and NPSV-deep (Linderman et al., 2024) is a SV genotyping tool developed based on deep learning algorithms and these tools can detect, genotype and filter a wide range of SV types in WGS data.

However, there are limitations and drawbacks associated with each tool, for example, tools with high precision could have missed true positives and tools with higher specificity could result in many false positives. Some tools require higher computational power and memory, and some tools may struggle in repetitive genomic regions to detect SVs leading to inaccurate results. Therefore, determining the optimal tools for a specific study remains a challenge both computationally and scientifically. Since each of these tools has its own strengths and weaknesses, there is no gold standard tool that can capture all the types of SVs with higher accuracy (Tattini et al., 2015). Hence, the detection of SVs requires integrated and reproducible strategies, the selection of SV detection tools should rely on the data types, study requirements, complexity of the genome and the type of SVs of interest (Mahmoud et al., 2019; Joe et al., 2024). Also, as many studies have suggested, for more comprehensive SV detection use of a consensus or ensemble approaches with complementary evidence types would be the best practice as it combines the capabilities of various tools to improve overall accuracy (Kosugi et al., 2019; Yang, 2020; Joe et al., 2024). For example, using tools that use RD strategy to detect copy number changes, SR for precise break points and RP for insertions and inversions, could offer more deeper insights into complex SVs rather than using multiple callers that use the same strategy (Kosugi et al., 2019; Tattini et al., 2015). Also, merging these outputs requires careful considerations, such as confidence thresholds (support from more than 2 callers or evidence types) and categorizing these calls into high confidence based on multiple signal support (Liu et al., 2024). For more accurate results, validation strategies should also be orthogonal and specifically matched to SV type and size; small SVs (<1 kb) are best validated by PCR or Sanger sequencing, while medium SVs (1–100 kb) are commonly validated by quantitative PCR, ddPCR, long read sequencing and optical mapping methods and larger complex SVs (>100 kb) are benefit from cytogenetics methods (FISH, karyotyping) or linked-read/Hi-C data (Ho et al., 2020). Furthermore, benchmarking with high confidence and well characterized truth sets (e.g., HGSVC, GIAB) and report precision, recall, breakpoint accuracy and genotype concordance stratified by SV type, size and genomic context (including repeat rich regions, telomeric and centromeric regions as these often possess particular challengers for accurate SV detection) are essential to reveal systematic biases, to assess method performances and to better understand the strengths and limitations (Liu et al., 2024). Pipelines should also include pre-filtering (based on minimum read support, quality thresholds) and post-processing (genotype refinement and annotation using population databases such as gnomAD-SV) to provide a rigorous and reproducible framework for SV discovery. By following these best practices, and documenting all software versions, parameters and reference genome build, can make SV calling workflows more robust and reliable, ensuring reproducibility and transparency. Also, use of new genome assemblies like pangenome and the use of artificial intelligence could enhance the accuracy and reliability of SVs detection, helping to refine methods, improve consistency and meaningful comparison in SV research across different datasets and platforms thereby improving our understanding of the genetic underpinning of CVD which could facilitate improved risk assessment strategies. While caller performance is central to accurate SV discovery, population-scale interpretation requires additional considerations related to genotyping, allele-frequency estimation, harmonization, and statistical modelling.

## 4 Structural variants and applied genomic epidemiology

SVs are increasingly recognized as an important contributor to disease risk and genetic variation in recent genomic studies, and their analysis strategies differ across population studies. Genotyping of SVs includes the representation of SVs as either presence/absence, which indicates whether a variant is present or absent in an individual’s genome (e.g., a deletion may present in a population while it is absent in another) or muti-allelic copy number, which provides more detailed information on quantitative copy number dosage of a specific SV (e.g., a duplication may have a 2 or more copy number in different individuals within a population) (Ho et al., 2020). These could provide insights to researchers on how the degree of variation of SVs affects different populations. Allele frequencies estimate the frequency of an allele/variant within a population, which is crucial for understanding the genetic diversity and evolutionary dynamics of populations. These estimations for SVs can be challenging as larger sample sizes are required to improve power and accuracy of rare variants that are specific to individuals or continental groups (Sudmant et al., 2015). Also, handling of population stratification is important as it can lead to biased results due to ancestry, geographical location or other factors. Use of approaches like principal component analysis (PCA) to group individuals with similar genetic backgrounds and use of mixed linear models to adjust for confounding ancestry effects can improve the accuracy and reliable interpretation of SVs and their role in complex diseases (Sudmant et al., 2015; Price et al., 2006). For association testing, common SVs are mostly analysed using regression models similar to those used in SNP-based GWAS, which involve linear or logistic regression models adjusted to covariates such as sex, ancestry PCAs and batch effects, particularly in large cohorts like UK Biobank (Collins et al., 2020). In contrast, rare SVs lack sufficient power for individual testing; therefore, they are often studied through burden tests, which combine many rare variants in a gene or regulatory region to improve statistical power. Studies may use different SV calling pipelines that often vary in sensitivity and breakpoint resolution, reference genomes, which could introduce errors in repetitive regions and batch effects, which can introduce inconsistencies due to different sequencing methods, read lengths and coverage differences leading to systemic bias and reduce power across studies. To overcome these problems, harmonization across cohorts remains a significant challenge, strategies like the use of unified pipelines, careful lift-over coordinates, standardized quality control and consistent annotations are essential for reliable cross-cohort SV analyses. Furthermore, explicit integration of SV information with SNP-based polygenic or genetic risk scores (PRS/GRS) can improve the risk prediction models (Kullo, 2025). The increment in the prediction value of these models by incorporating the SV data can be estimated using metrics such as area under the curve (AUC), C-index and net reclassification improvement (NRI), with performance validated in independent cohorts to ensure generalizability and reliability. This approach can significantly refine risk stratification for certain individuals, highlighting the integrated value of common and rare genetic variants in predictive modelling. Insights from population-level analyses directly inform clinical implementation, where precise SV resolution and interpretation are essential for diagnosis, risk stratification, and therapeutic decision-making.

## 5 Whole genome sequencing and cardiovascular genomic medicine

In clinical diagnosis, precise detection of SVs and their breakpoints are important to determine the specific genes impacted by an SV and understand its functional consequences (Leung et al., 2022). Genetic tests that identify these variants in pre-symptomatic individuals could serve as a critical tool that enables timely intervention and improved patient outcomes. The current clinical diagnostic analysis based on WES is largely restricted to the identification of SNPs and small indels in the coding regions which is about 1% of the genome. The application of WGS for the identification of potentially causal SVs may lead to improved diagnostic accuracy, especially in patients with negative WES screening. The study of Pagnamenta et al. showed that SVs contribute significantly to the diagnostic yield of clinical WGS for rare diseases and incorporation of WGS data to facilitate comprehensive analysis of the entire genome could increase clinical diagnostic yield in previously unsolved WES cases (Pagnamenta et al., 2023). Stranneheim et al. have also shown that the detection of SVs in WGS data has increased their diagnostic yield by 7.5% and they were able to achieve 19%–54% case solve rates in clinical WGS across a broad areas of disease types (Stranneheim et al., 2021). Advancements in WGS have improved the detection of SVs that are associated with inherited CVD although the number of available genetic tests are limited due to the incomplete understanding of the genetic basis for CVD (Marian, 2020). Furthermore, WGS data has the capability to understand the individuals’ SV profiles which could lead to improved personalized treatment strategies and therapeutic targets that allow efficient prompt execution of personalized treatment plans. Additionally, the identification of SVs associated with CVD based on gender and diversity of ethnicity could enable more accurate risk stratification thereby improving the precise diagnoses, preventive measures and patient outcomes. Despite these advances, several technological and analytical limitations still constrain the full integration of SVs into cardiovascular genomics, motivating continued innovation.

## 6 Future directions

Over the past 10 years, SVs have emerged as an important type of genomic variant as they can affect larger areas of the genome potentially contributing to underlying genetic causes of many complex diseases. More investigations are now required to understand the mechanisms involved in how SVs affect gene regulation, expression, their contributions to phenotypic traits and susceptibility to complex diseases. There is now an opportunity to identify the types of SVs in addition to CNVs that are involved in complex diseases like CVD. When detecting and identifying the clinical importance of SVs, the availability of sophisticated data analytical and interpretation tools along with comprehensively annotated databases have been major bottlenecks. Ongoing research and developments are continuously improving the accuracy and efficiency of SV detection tools. With these improvements, the generation of a standardized method to detect SVs will be important for precise identification of SVs ensuring consistency and reproducibility in research and clinical applications. Emerging technologies such as high-fidelity long-read sequencing have the capability to accurately detect SVs present in repetitive and complex regions that short reads often miss or struggle to read, thereby reducing the chances of false positives and allowing precise breakpoint resolution. Precise breakpoint identification could reveal the mechanisms involved in SV formation, facilitating the identification of SV hotspot loci linking to CVD. Development of high-quality genome assemblies and pangenome efforts to represent the genetic diversities will help in better resolving complex SVs overcoming the single linear reference bias and improving the SV calling across ancestries and accurate genotyping of population-specific or rare SVs. This could enhance the cross-cohort comparisons in cardiovascular genetics studies and help to distinguish pathogenic SVs from benign population variants, improving more equitable and accurate cardiovascular risk assessment across diverse populations. Additionally, the integration of multi-omics data such as transcriptomics, proteomics, epigenetics and functional studies to develop comprehensive databases to annotate SVs could facilitate functional interpretation linking the SVs to downstream molecular consequences such as integration of SVs and RNA-seq data could identify the effects on gene expression, and splicing in different cardiac disease relevant cell types, SVs and epigenomics data could reveal how they disrupt the regulatory regions active in cardiac cells and proteomics could link SVs to altered protein abundance or metabolic pathways that lead to different cardiac phenotypes. Also, expanding the diversity of study populations provides the opportunity to enhance the identification and interpretation of SVs. These advancements will pave the way for better understanding and detection of SVs, improve genotype-phenotype mapping for complex cardiovascular traits which will ultimately help future research to fully uncover the role of SVs in diseases like CVD and improve clinical management strategies.

## 7 Conclusion

SVs are now recognised as a major, yet historically under-detected, contributor to CVD. Recent advances in WGS, including short-read calling and tools along with long-read technologies, have transformed our ability to detect, interpret and clinically apply SVs in research and diagnostic settings. Despite this progress, important challenges remain, including improved breakpoint resolution, harmonisation across cohorts, robust functional annotation, and the integration of SVs into risk-prediction and clinical decision-making.

As analytical methods mature and population-scale datasets increasingly incorporate SV information, the field is poised for a step change in understanding the genetic mechanisms underlying CVD. Continued refinement of SV calling algorithms, stronger benchmarking standards, expansion into diverse populations, and integration with multi-omics and pangenome references are essential to realise their full potential. Achieving consistent, accurate and clinically interpretable SV detection will enable more precise genomic diagnoses, improved risk stratification, and better-targeted prevention and treatment strategies for CVD.
